# Immunohistochemical Analysis of Vimentin in Oral Submucous Fibrosis

**DOI:** 10.1155/2013/549041

**Published:** 2013-06-11

**Authors:** Meghanand T. Nayak, Anjali Singh, Rajiv S. Desai, S. S. Vanaki

**Affiliations:** ^1^Department of Oral & Maxillofacial Pathology, Vyas Dental College and Hospital, Jodhpur, Rajasthan 342011, India; ^2^Department of Oral Medicine & Radiology, Vyas Dental College and Hospital, Jodhpur 342011, India; ^3^Department of Oral & Maxillofacial Pathology, Nair Hospital Dental College, Mumbai 400008, India; ^4^Department of Oral & Maxillofacial Pathology, PMNM Dental College & Hospital, Bagalkot 587101, India

## Abstract

*Background*. Oral submucous fibrosis (OSF), a precancerous condition, is characterized by abnormal accumulation of collagen fibers in oral submucosa. Vimentin is a Class 2 intermediate filament (IF) and primarily expressed in cells of mesenchymal origin. Vimentin is also found to be involved in cell growth, cell cycling, and tumour differentiation. *Objective*. The purpose of the study was to compare the expression of vimentin in various histological grades of OSF. *Materials and Methods*. To assess the immunohistochemical expression of vimentin in 20 mild cases of OSF, 20 severe cases of OSF, and ten cases of normal oral buccal mucosa. *Results*. The overall staining intensity of vimentin significantly increased statistically (*P* < 0.01) in OSF cases over normal control. A significant increase in the staining intensity of vimentin was also noted in the fibroblasts of severe cases of OSF (*P* = 0.03). *Conclusion*. Considering the marked vimentin expression in the present study, future studies should include cytoskeleton IF and other filaments in the fibroblasts of OSF.

## 1. Introduction

The areca nut (AN), popularly known as “betel nut,” is one of the oldest known masticatories among Indians and it is estimated that around 600 million people around the world use AN [1]. The oral health consequences of chewing AN are varied. Among its many effects on oral structures, of significance is the development of oral submucous fibrosis (OSF)—a potentially malignant condition [2].

OSF is characterized by abnormal accumulation of collagen fibers in oral submucosa. Experimental studies have shown that ethanolic extracts of the AN stimulate collagen synthesis in human dermal fibroblasts [3]. AN extracts may also stabilize collagen fibrils and render them resistant to degradation. In comparison with normal fibroblasts, OSF fibroblasts synthesized larger amounts of collagen; they have higher procollagen mRNA levels; and they produce type I collagen trimer, which is resistant to degradation [4].

To date, there has been little research exploring the possible effects of arecoline on the cytoskeleton components [5, 6]. The vimentin antibody recognizes a 57 kDa IF and labels a variety of mesenchymal cells, including melanocytes, lymph cells, endothelial cells, and fibroblasts [7]. Vimentin expression is significantly enhanced in cell growth, cell cycling, tumor differentiation, and during the process of tumorigenesis [5]. Although OSF is regarded as a precancerous condition, the extent of vimentin expression in human buccal mucosa in the presence of arecoline is not clearly understood. 

The aim of the study was to compare the expression of vimentin in various histological grades of OSF and relating it to the normal mucosa. Further, an attempt was made to elucidate the possible role of vimentin in the pathogenesis of OSF.

## 2. Subjects and Methods

The study was carried out with a total number of 40 patients who visited the authors' institute and were diagnosed as having OSF both clinically and histologically. The consent was collected from all patients and approved by the Institutional Review Board. The histopathological evaluation and grading of the OSF cases were performed with the criteria given by Pindborg and Sirsat [8]. Ten samples of normal oral mucosa (buccal mucosa) procured from age- and sex-matched subjects constituted the controls. 

### 2.1. Hematoxylin and Eosin Staining

Formalin-fixed paraffin-embedded specimens were subjected to four *μ*m thick sections and were stained with hematoxylin and eosin for histologic confirmation of clinical diagnosis and other evaluations. These sections were used for grading of OSF. 

### 2.2. Immunohistochemistry Protocol

 Eight-micron-thick sections were taken on poly-L-lysine (PLL) slides and stained with monoclonal mouse antivimentin antibody (Vim 3B4, 1 : 200; Dakopatts CA, USA) using a standard avidin-biotin-peroxidase complex method. Enzymatic predigestion with proteolytic enzymes (Proteinase K) was done for greater staining intensity and uniformity on formalin-fixed tissue sections. Diaminobenzidine (DAB, Zymed) was then used as the substrate for localizing the antibody binding. The preparations were counterstained with the Harris hematoxylin, mounted with neutral mounting medium and examined under light microscope for immunoperoxidase reactivity. Positive staining of tonsil tissue was considered as positive control, while negative staining of epithelial tissue was considered as negative control for vimentin staining.

#### 2.2.1. Scoring

The vimentin-stained sections were studied in detail by three oral pathologists independently and the tissue sections were graded as follows. 

The sections were viewed initially in low power and the connective tissue stroma was divided into two zones—the superficial zone and deep zone. Each zone was considered separately and scored on a scale of 0–3 with 0 indicating no staining, 1 indicating mild staining, 2 indicating moderate staining, and 3 indicating intense staining. 

The tissue sections were later viewed under high power and the cellular localization of the staining was studied. The staining for fibroblasts and endothelium was considered separately and scored on a scale of 0–3 with 0 indicating no staining, 1 indicating mild staining, 2 indicating moderate staining, and 3 indicating intense staining. 

The overall intensity of staining was noted in all the cases and scored on a scale of 0–4 with 0 indicating no staining, 1 indicating mild staining, 2 indicating moderate staining, 3 indicating intense staining, and 4 indicating the most intense staining. 

### 2.3. Statistical Analysis

Descriptive data that included frequency (number of cases with score 0, 1, 2, 3, and 4), mean, and standard deviation were determined for all the groups. Since the assessment of staining was done based on the scores, a nonparametric method, the Mann-Whitney test, was used for pairwise comparisons. Interobservers' precision was assessed by computing the mean scores between the three observers simultaneously by using one-way ANOVA for multiple group analysis. For all the tests, a *P* value of less than or equal to 0.05 was considered statistically significant. All the calculations were done on a personal computer using Minitab software (USA, v.16).

## 3. Results

**Figure 1:**
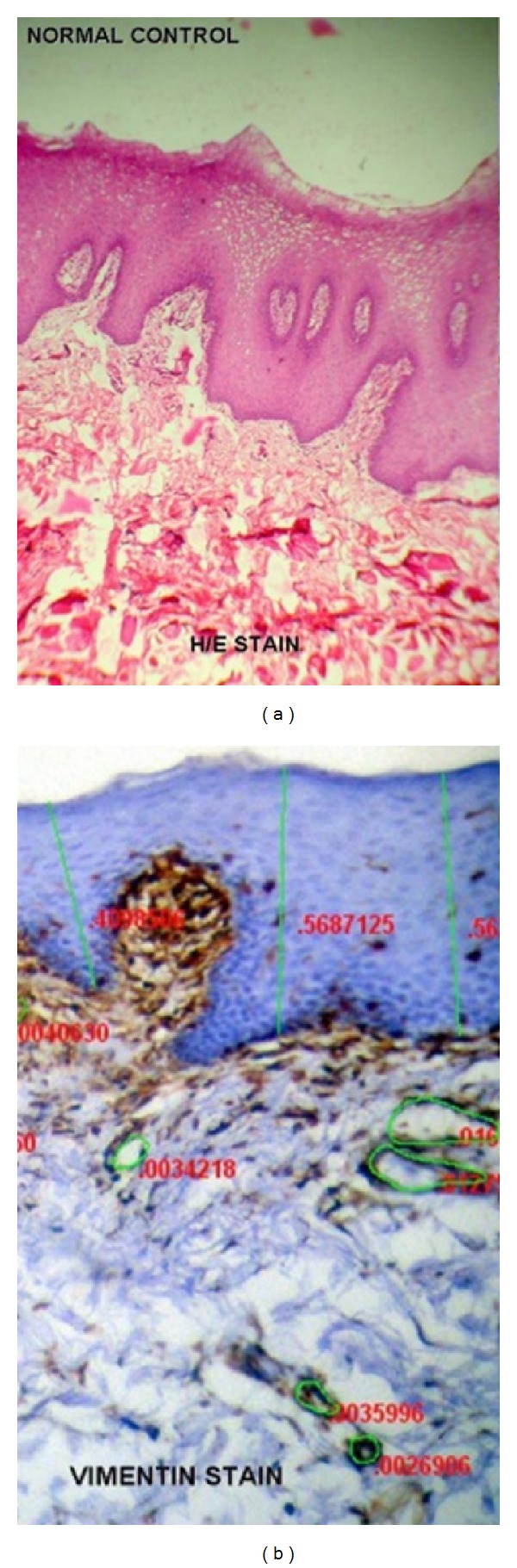
(a) Normal buccal mucosa under H/E staining. (b) Normal buccal mucosa under vimentin immunohistochemical staining. Vimentin is expressed in most cells of mesenchymal origin, including fibroblasts, endothelial cells.

**Figure 2:**
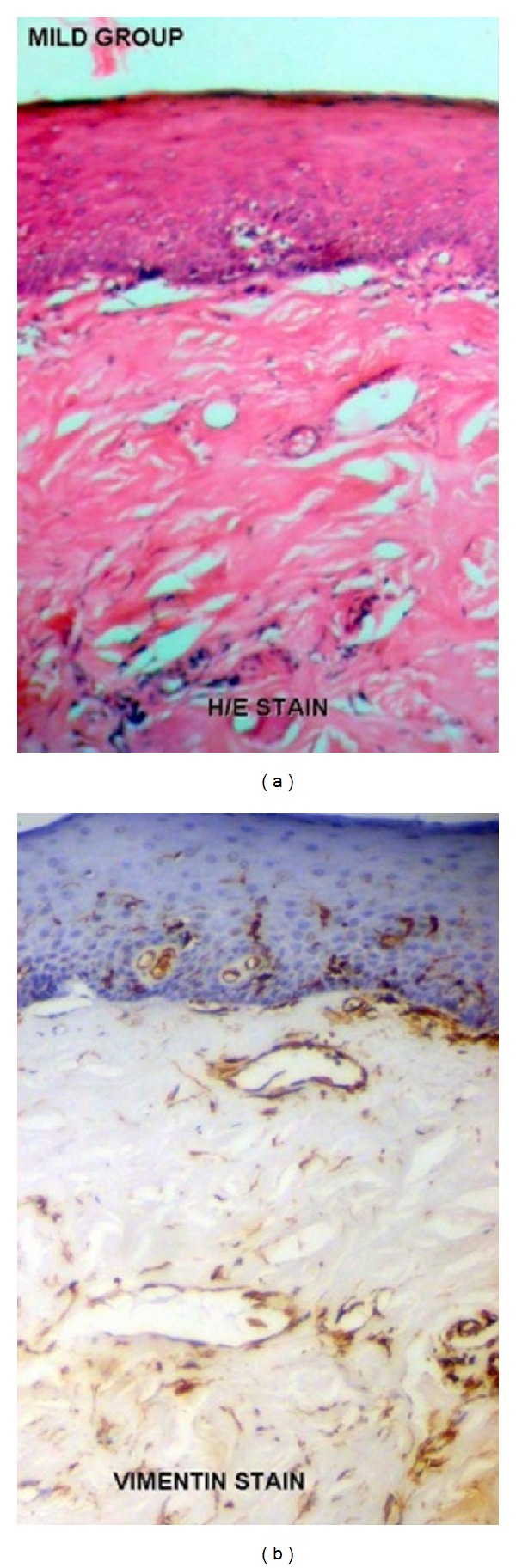
(a) Mild cases of Oral submucous fibrosis under H/E staining. (b) Mild cases of oral submucous fibrosis under vimentin immunohistochemical staining. Note the staining intensity of vimentin in endothelial cells is markedly increased as compared to normal subjects.

**Figure 3:**
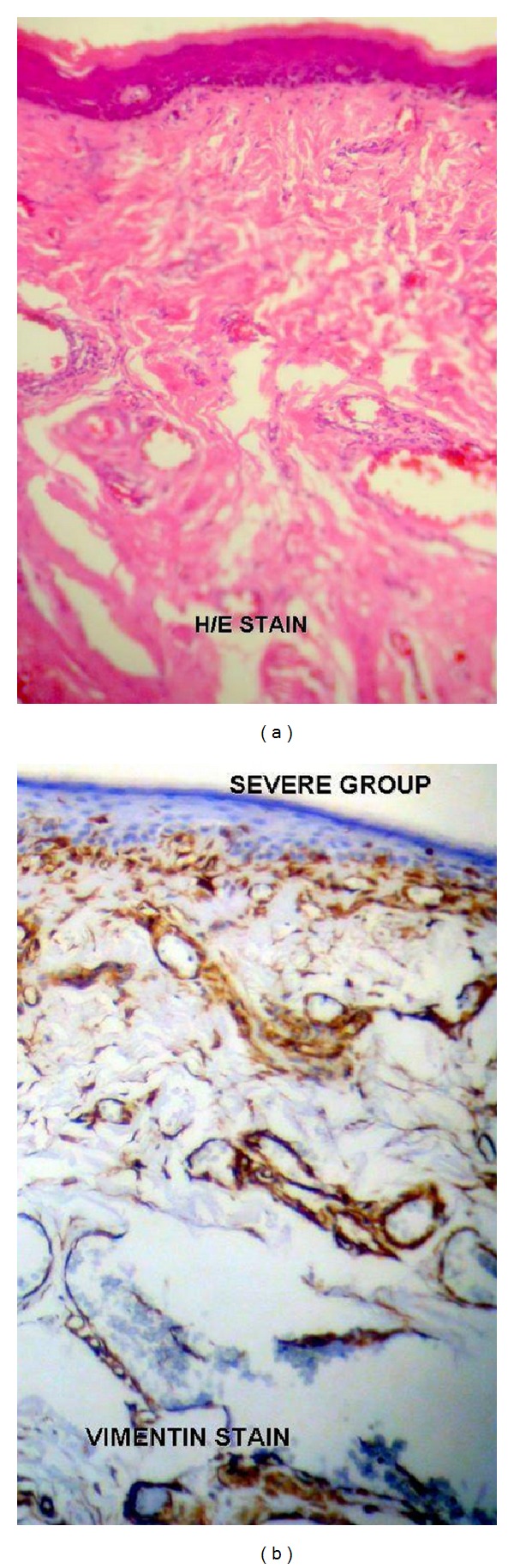
(a) Severe cases of oral submucous fibrosis under H/E staining. (b) Severe cases of oral submucous fibrosis under vimentin immunohistochemical staining. Note the intense vimentin staining in the subepithelial zone and the deeper zone of the connective tissue stroma.

A total sample size of 50 cases was included in the study. Forty cases of OSF constituted the study group and ten cases of normal (Figure 1) were taken as controls. The age ranged from 18 to 45 years in OSF cases and the mean age was 26.9 ± 8.1. Out of total ten cases of normal controls, the age range was 20 to 37 years while 24.9 ± 5.1 was the mean age. Of the total 40 cases, 10% (4) of the cases were in very early stage, 40% (16) of cases were in early stage, 40% (16) of cases were in moderately advanced stage, and 10% (4) of the cases were in advanced stage of the disease. The very early-stage cases and early-stage cases were clubbed together and considered as mild group totaling 20 cases of the disease (Figure 2). The moderately advanced and advanced cases were combined and constituted the severe group totaling the remaining 20 cases (Figure 3). The analysis to compare staining pattern in different groups was done. The distribution scores of the vimentin staining in various localizations and overall intensities are represented in the Tables 1, 2, 3, 4 and 5.

The intensity of staining was scored systematically on different areas of the connective tissue; that is, the superficial/subepithelial zone and the deep zone were considered separately. The individual cell stained was also noted and the pattern of staining intensity was graded separately for the fibroblasts and endothelium. These gradings were made by one pathologist and two oral pathologists individually. The scores were later compared for interobservers' confidence and a very high confidentiality was recorded which shows the method of scoring is reliable. The scores were graded on a scale of 0–3 for the tissue localization (superficial and deep) and cell localization (fibroblasts and endothelium), whereas a score on scale 0–4 was recorded for overall intensity. 

The superficial scores for mild cases had a mean of 1.8 with a standard deviation of 1.1 while the scores in severe cases had mean average of 1.9 and standard deviation of 1.1. The normal cases did not show any intense staining with score of 3 but had a mean score of 1.2 with deviation of 0.8. The Mann-Whitney test showed no significant difference between mild versus severe cases, mild versus normal cases, and severe versus normal cases.

The deep scores for mild cases were 1.8 ± 0.9 while the scores in severe cases had mean average of 2.1 and standard deviation of 0.7. The normal cases did not show any intense staining and had a mean score of 1.2 with deviation of 0.8. The significant difference in the *P* value was noted between severe and normal cases while as rest of the groups did not show any significant differences.

The staining patterns in the fibroblasts were recorded and a score of 2.3 mean was noted in mild cases of OSF while 2.4 was scored for severe cases which was significantly more than the normal cases' score of 1.5. The *P* value calculated for severe cases and normal is statistically significant with a score of 0.03. 

The endothelium showed an intense staining pattern in mild cases and severe cases of OSF but no cases showed intense staining in normal cases. The mean score of 2.5 was recorded in mild cases, 2.7 in severe cases and 1.4 in normal cases. The *P* value was very significant between mild versus normal and severe versus normal cases.

The pattern of overall intensity was scored on a scale of 0 to 4. The mild cases showed the intensity pattern of 3 in ten cases and 4 in four cases with a mean average of 2.7 and a standard deviation of 1.2. The severe cases showed intense staining with score 3 in eight cases and score 4 in ten cases averaging 3.2 with deviation of 1.2. The normal cases showed no intense staining in any of the cases and showed only a score of 2 in 14 cases. The mean scores for normal cases were 1.5 with variation of 0.8. The *P* value was very significant in mild cases versus normal and severe cases versus normal. 

## 4. Discussion

The pathogenesis and treatment of OSF have been a subject of controversy, ever since Schwartz [9] first described the condition in 1952. In 1980, Pindborg had estimated about 250,000 OSF cases in India, but the estimation in 2002 had increased to 2 million cases, an eightfold increase in the disease [9]. With increasing use of gutka/pan masala products in India, the number of OSF cases has drastically increased. OSF is a peculiar disease which is considered as a premalignant condition; however it demonstrates a variable biological behavior and the response to any treatment is still a disheartening one. In recent years several epidemiological studies have highlighted the etiological role of AN in OSF. 

Arecoline, the major alkaloid of the AN, has been reported as one of the causative factors for the chromosomal aberrations in OSF patients [10]. Tissue culture experiments using human fibroblasts obtained from OSF subjects revealed an elevation of collagen synthesis by 170% when compared to control cultures [3]. It has been demonstrated that arecoline and arecaidine promote collagen formation [11]. Stabilization of collagen and prevention of collagenase degradation in oral mucosa and attendant increase of lysyl oxidase activity contribute to the extra cellular matrix component accumulation in OSF [12]. 

The cytoskeleton of most vertebrate cells consists of microfilaments, microtubules, and IF. IF plays a supporting or general structural role [13]. Vimentin is a Class 2 IF, which is primarily expressed in cells of mesenchymal origin and is the most abundant IF [14]. In dental tissues, it has been immunolocalized in fibroblasts of the periodontal ligament and dental pulp, in odontoblasts and in fibroblasts of the dental papilla and dental follicle during tooth development [15]. Proposed function of vimentin includes regulation of cell attachment, subcellular organization and signal transduction from the plasma membrane to the nucleus. It has been suggested that vimentin expression in vitro is a sign of dedifferentiation [16, 17].

Studies have shown that arecoline induces collagen formation, demonstrates cytotoxicity and also stimulates double-stranded nucleic acid synthesis [10] and cell morphology change. This change in the cell morphology implicates arecoline in cytoskeletal disturbance associated with interference in cell mitosis and intracellular transport mechanisms. The effect of arecoline on the vimentin in normal human buccal mucosal fibroblasts has been studied previously by very few authors.

Antivimentin antibody has been used previously to study the intensity of staining in the OSF cases [5]. The change in the intensity of staining in the arecoline-induced OSF is not clearly recorded and localized according to the individual tissue or the cell staining. Thus the present study aimed to note the expression of the vimentin in normal human buccal mucosal fibroblasts and compared it with the high expression in the fibroblasts of various histological grades of OSF patients using immunohistochemical methods. This study also aimed at elucidating the possible role of vimentin in the pathogenesis of OSF.

The antivimentin antibody (clone: Vim 3B4) was used in our present study for immunohistochemical staining. This clone was preferred over the clone V9, which was used by other researchers [5], as Vim 3B4 clone has an added advantage that it stains well on the formalin-fixed tissue sections [7]. This procedure also involved usage of Proteinase K for enzyme predigestion or the antigen retrieval. The staining pattern obtained in our study was more uniform and greater stain intensity was noticed. 

The connective tissue from normal buccal mucosa consists of loosely woven collagen bundles in the lamina propria revealing a fine reticular pattern next to the epithelium and a coarser pattern deeper in the lamina propria. Vimentin gets labeled in the collagen fibers slightly and more predominantly around the inflammatory cells [7].

Significant difference was noted in the fibroblasts staining between the scores of normal and OSF cases. The difference could however be the result of the presence of a subtype of fibroblast which is more susceptible to external stimulation or gene modulation. This suggests the increase in the intensity pattern in OSF cases in our study is similar to the other studies [5]. These data suggest the potential involvement of vimentin in the pathogenesis of OSF. Although the full significance of these findings remains to be elucidated, the study indicates that the expression of IF in OSF cases is a complex phenomenon. IF is believed to play a primarily structural role within the cell. Expression of vimentin appears to be closely related not only to type and stage of differentiation but also to basic properties of cellular kinetics and contact [13]. We suggest the results of our study may advance the understanding of the possible pathogenesis of OSF.

## 5. Conclusion

The pathogenesis and occurrence of the disease are still an unsolved mystery. The disease is an excellent model to study pathological fibrosis and has a similar histological picture to other pathological fibrosis elsewhere in the body. The need of the hour is to develop a treatment modality to treat the disease successfully and this can be achieved only by understanding the pathophysiology of the disease process. Research pointing towards the study of intracytoplasmic organelles should be considered along with the study of cytoskeleton components of the fibroblasts. The function of vimentin in cultured cells, and of cells in vivo, remains obscure. Further research is required for detecting vimentin gene transcripts specifically and to know whether OSF evolves solely as a result of increased altered de novo synthesis and deposition of vimentin by AN constitutes. Considering the evident vimentin expression in the present study, future studies should also include cytoskeleton IF and other filaments and this might prove to be a potential therapeutic target for OSF. 

## Figures and Tables

**Table 1:** Pattern of vimentin scores at superficial localization in mild OSF cases, severe OSF cases, and control group.

	Group	No.	Scores (0–3)				Mean ± SD	Mild v/s severe	Mild v/s normal	Severe v/s normal
			0	1	2	3				
Superficial	Mild	20	2	8	2	8	1.8 ± 1.1	*P* = 0.87 (N.S)	*P* = 0.25 (N.S)	*P* = 0.15 (N.S)
	Severe	20	2	6	4	8	1.9 ± 1.1			
	Normal	10	2	4	4	—	1.2 ± 0.8			

**Table 2:** Pattern of vimentin scores at deep localization in mild OSF cases, severe OSF cases, and control group.

	Group	No.	Scores (0–3)				Mean ± SD	Mild v/s severe	Mild v/s normal	Severe v/s normal
			0	1	2	3				
Deep	Mild	20	2	4	10	4	1.8 ± 0.9	*P* = 0.51 (N.S)	*P* = 0.14 (N.S)	*P* < 0.05 (S) [0.03]
	Severe	20	—	4	10	6	2.1 ± 0.7			
	Normal	10	2	4	4	—	1.2 ± 0.8			

**Table 3:** Pattern of vimentin scores of fibroblasts in mild OSF cases, severe OSF cases, and control group.

	Group	No.	Scores (0–3)				Mean ± SD	Mild v/s severe	Mild v/s normal	Severe v/s normal
			0	1	2	3				
Fibroblasts	Mild	20	2	2	4	12	2.3 ± 1.1	*P* = 0.93 (N.S)	*P* = 0.06 (N.S)	*P* < 0.05 (S) [0.03]
	Severe	20	2	—	6	12	2.4 ± 1.0			
	Normal	10	2	2	5	1	1.5 ± 1.0			

**Table 4:** Pattern of vimentin scores of endothelium in mild OSF cases, severe OSF cases, and control group.

	Group	No.	Scores (0–3)				Mean ± SD	Mild v/s severe	Mild v/s normal	Severe v/s normal
			0	1	2	3				
Endothelium	Mild	20	2	—	4	14	2.5 ± 1.0	*P* = 0.36 (N.S)	*P* < 0.01 (V.S) [0.006]	*P* < 0.01 (V.S) [0.0011]
	Severe	20	2	—	—	18	2.7 ± 0.9			
	Normal	10	2	2	6	—	1.4 ± 0.8			

**Table 5:** Pattern of overall intensity of vimentin scores in mild OSF cases, severe OSF cases, and control group.

	Group	No.	Scores (0–4)					Mean ± SD	Mild v/s severe	Mild v/s normal	Severe v/s normal
			0	1	2	3	4				
Overall Intensity	Mild	20	2	—	4	10	4	2.7 ± 1.2	*P* = 0.17 (N.S)	*P* < 0.01 (V.S) [0.007]	*P* < 0.01 (V.S) [0.002]
	Severe	20	2	—	—	8	10	3.2 ± 1.2			
	Normal	10	2	1	7	—	—	1.5 ± 0.8			
